# Draft Genome Sequences of Two Clinical Isolates of *Lactobacillus rhamnosus* from Initial Stages of Dental Pulp Infection

**DOI:** 10.1128/genomeA.00073-12

**Published:** 2013-01-15

**Authors:** Zhiliang Chen, Marc R. Wilkins, Neil Hunter, Mangala A. Nadkarni

**Affiliations:** a Systems Biology Initiative, School of Biotechnology and Biomolecular Sciences, the University of New South Wales, New South Wales, Australia; b Institute of Dental Research, Westmead Centre for Oral Health and Westmead Millennium Institute, Westmead, Australia; c Faculty of Dentistry, the University of Sydney, Sydney, Australia

## Abstract

Here we report the draft genomic sequences of two clinical isolates of *Lactobacillus rhamnosus* from infected dental pulps representing the initial stages of infection of pulp tissue. Based on 454 FLX+ pyrosequencing, the two clinical isolates infecting vital pulp had a genome length of 2.9 Mbp with distinct genomic signatures.

## GENOME ANNOUNCEMENT

Lactobacilli colonize multiple niches in the human body. Lactobacilli with probiotic properties are predominantly represented in the human gastrointestinal microflora and the healthy vaginal microflora. Many strains of various species of lactobacilli are “generally regarded as safe (GRAS)” bacteria that possess probiotic benefits and are therefore not considered as pathogens (1). *Lactobacillus rhamnosus* GG (ATCC 53103) is widely used and has been extensively studied in probiotic applications. However, in addition to the beneficial effects of lactobacilli, strains of *L. rhamnosus* and *Lactobacillus paracasei* have been detected as pathogens in infective endocarditis (2). Similarly, *L. rhamnosus* with a different pulsed-field gel electrophoresis (PFGE) pattern from that of *L. rhamnosus* GG was reported to cause meningitis (3). Diverse lactobacilli have been consistently implicated in the initiation and progression of dental caries, the most common disease of the oral cavity (4, 5). Fluorescence *in situ* hybridization (FISH) analysis has provided insights into the community structure of polymicrobial consortia in infected tissue microbiomes and the precise identification of lactobacilli that are prominent in the initial stages of infection of dental pulp (6).

Draft genome sequences of two *L. rhamnosus* strains, LRHMDP2 and LRHMDP3, have been generated. Both *L. rhamnosus* clinical isolates were sourced from infected dental pulps from carious teeth categorized as representing the initial stages of infection of pulp tissue. Genomic DNA was sequenced by a whole-genome shotgun strategy using Roche 454 GS (FLX +) pyrosequencing at the Ramaciotti Centre for Gene Function Analysis, University of New South Wales. A total of 77,329 reads from LRHMDP2 and 75,985 reads from LRHMDP3 were generated to reach a depth of ~17-fold genome coverage, and assembled by a Newbler assembler 2.6 into 51 and 47 contigs longer than 200 bp, respectively. Gene definition and annotation of these two strains were performed by merging the result from the RAST (Rapid Annotation using Subsystem Technology) server (7) and tRNAscan-SE (8).

The sequences of the two strains, *L. rhamnosus* LRHMDP2 and *L. rhamnosus* LRHMDP3, include 2,911,290 bp and 2,911,934 bp, respectively. The genomic sequence of LRHMDP2 is comprised of 2,910 coding sequences (CDSs), 7 rRNA loci, and 56 tRNAs, with a G+C content of 46.6%. In comparison, the genome sequence of LRHMDP3 contains 2,925 CDSs, 6 rRNA loci, and 57 tRNAs with a G+C content of 46.6%.

Comparative genomic analysis of LRHMDP2 and LRHMDP3 with a probiotic *L. rhamnosus* GG (ATCC 53103) showed the absence of exopolysaccharide (*eps*) and *spaCBA* pilus clusters in both of the clinical isolates. New genes confined to the clinical isolates included a modified *eps* cluster, a two-component sensor histidine kinase, a response regulator, and ABC transporters for ferric iron, RNA polymerase sigma 54 factor RpoN, MutR, NtrC, ArsR and zinc-binding Cro/CI family transcriptional regulators. A ThiJ**/**PfpI family protein and phosphotransferase system (PTS) system mannose, galactitol, mannitol, cellobiose, and beta-glucoside-specific II components were also identified.

### Nucleotide sequence accession numbers.

This Whole Genome Shotgun project has been deposited at DDBJ/EMBL/GenBank under the accession numbers AMQW00000000 for *L. rhamnosus* LRHMDP2 and AMQX00000000 for *L. rhamnosus* LRHMDP3. The version described in this paper is the first version, AMQW01000000 and AMQX01000000.
